# A qualitative investigation of healthcare workers’ strategies in response to readmissions

**DOI:** 10.1186/s12913-018-2945-9

**Published:** 2018-02-27

**Authors:** Priyadarshini R. Pennathur, Brennan S. Ayres

**Affiliations:** Department of Mechanical and Industrial Engineering, University of Iowa, Iowa City, 52242 USA

**Keywords:** Hospital readmissions, Grounded theory, Qualitative research, Healthcare worker, Health information systems

## Abstract

**Background:**

Readmission of a patient to a hospital is typically associated with significant clinical changes in the patient’s condition, but it is unknown how healthcare workers modify their provision of care when considering these changes. The purpose of the present study was to determine how healthcare workers shift their care strategies when treating readmitted patients.

**Methods:**

A typical case sampling study of healthcare workers was conducted using the grounded theory approach. The study setting comprised several patient care units at an academic center and tertiary-care hospital. We purposively sampled 34 healthcare workers (19 women, 15 men) to participate in individual interviews, either face-to-face or by telephone. We asked the participants semi structured questions regarding their thoughts on readmissions and how they altered their process and behavior for readmitted patients. Interviews were audio-recorded and transcribed. We used a qualitative data analyses based on an inductive approach to generate themes about how healthcare workers shift their strategies for readmitted patients.

**Results:**

Healthcare workers’ shifts in strategy for readmissions were reflected in three major themes: clinical assessment, use and management of information, and communication patterns. Participants reported that they became more conservative in their assessment of the clinical condition of a readmitted patient. The participants also indicated that readmitted patients would be treated in a similar way to normal admission based on care requirements; however, somewhat paradoxically, they also expressed that having access to prior patient information changed the way they treated a readmitted patient.

**Conclusions:**

Although healthcare workers may exhibit a tendency to become more conservative with readmissions, readily available patient information from the previous admission played a large part in guiding their thinking. A more conservative approach with a readmitted patient, on its own, does not necessarily lead to improved documentation or better patient care.

**Electronic supplementary material:**

The online version of this article (10.1186/s12913-018-2945-9) contains supplementary material, which is available to authorized users.

## Background

Healthcare facilities constantly struggle with the tradeoffs associated with reducing costs [1–3], and improving patient safety [4–6] and care quality [7–9]. Readmissions, in particular, increase healthcare costs [1–3] and infection risks [4]. Consequently, hospitals use readmission rates as a quality metric [10–14]. The extent to which readmissions affect quality of care for the readmitted patient is however unclear. Gorodeski et al. [11], for example, reported higher readmission rates, but lower mortality among heart failure patients, raising questions on the impact of readmissions on quality of care.

Readmissions typically occur due to complexities in patients’ clinical conditions [13, 15], communication lapses between healthcare workers and patients [5, 16], and patients’ socioeconomic conditions [17, 18]. While some patients have a reasonable chance of improving their clinical condition after their first hospital visit, other patients either have serious medical conditions that can only be temporarily alleviated, or have co-morbidities that can cause them to be readmitted, indicating the complexities in readmissions due to patients’ clinical conditions [13, 15, 19, 20]. Additionally, the quality of communication between healthcare workers, and communication with patients after discharge can determine readmission rates [5, 16]. Poor handoff communication is a major cause of medical errors [21–25]. Time spent for handoff communication is not only valuable for exchanging a patient’s current status, but also for planning for improving patient safety and preventing readmissions. Socioeconomics [17, 18, 26–29] have also been implicated as a factor in repeat admissions, possibly due to a lack of resources to maintain health, a lack of access [30], non-compliance and non-adherence to medications, and a lack of exercise and a healthy diet.

While the causes for readmissions vary, there is consensus that readmissions are not good for the healthcare system, the patient or the healthcare worker. Patients who are readmitted can become more prone to infections in the hospital setting [13, 31–33]. With continued, repeated and lengthy hospital stays, patients may become dependent on care provided in the hospital setting, which may not be an effective strategy for improving their long-term health.

Current literature has examined the reasons for readmissions [5, 13, 16, 17, 19, 28], the impact of readmissions on the healthcare system and the patient [4, 32], and has reported clinical and administrative strategies to prevent readmissions [34–37]. However, understanding readmissions from the perspective of the healthcare workers who are at the frontline of patient care is limited [27, 38, 39]; in particular, research on any shifts in strategies that healthcare workers may make to treat readmitted patients is lacking.

Understanding healthcare worker shifts in care strategies when a patient is readmitted is important given its implications for patient care, error prevention, and healthcare cost reduction [27]. When patients are readmitted, healthcare workers usually first determine any significant changes in the patients’ clinical condition. When treating readmitted patients, they may also have to correct problems from the first admission. However, when treating a readmitted patient, do healthcare workers shift their care strategies significantly? What are the characteristics of the care strategy shifts, and what insights do they provide about patient care for a readmitted patient? Investigating these questions can provide valuable insights on the design of care processes for readmitted patients.

## Methods

### Aim

The study’s primary aim was to determine the specific shifts in the care strategies of healthcare workers when treating readmitted patients.

### Design

Semi-structured interviews were conducted with healthcare workers in intensive care units (ICUs), inpatient floors and outpatient clinics in an academic hospital. We used a purposive, typical case sampling study [40] of healthcare workers. We asked about readmissions and developed codes and analyzed the responses inductively using the grounded theory approach [41, 42] to document healthcare workers’ shifts in their strategies when dealing with readmitted patients.

### Settings

We conducted the study at patient care units in a leading US academic center and tertiary-care hospital, including: the Cardiovascular, Medical, and Surgical ICUs; Medical Cardiology, General Medicine, Adult Surgical Specialty Services, Respiratory Specialty Care units; and Respiratory Work and Social Work Services and Outpatient Ambulatory Care Services departments. The hospital has more than 30,000 patients a year and a 700-bed capacity. Readmission rates for our study hospital are similar to the national rates [43].

### Participants

We recruited participants using flyers, presentations, and referrals. We did not use any participant inclusion or exclusion criteria, for this typical case sampling study design. Typical case sampling is a type of purposive sampling where participants are selected to be “typical”, “representative” or “normal” for a particular phenomenon, or for explaining a particular process [40]. Hence, we used this approach to select participants who are typical healthcare workers, representative of the healthcare processes or behaviors that may result from it.

We purposively sampled participants representing units in the typical patient trajectory. Based on feedback from unit coordinators about staffing schedules, we aimed for two to five study participants in each role within a unit. The threshold number of participants not only closely corresponded with the number of healthcare workers who were scheduled during the data collection, but also represented typical staffing levels within the units. We stopped recruitment for specific roles, when a preliminary examination of successive interview responses indicated data saturation according to grounded theory protocols [42].

A brief review of research articles on sample size, grounded theory and data saturation reveals that there are no universally agreed recommendations on determining sample size requirements for qualitative studies due to the large number of variations under which a qualitative study might be conducted [42, 44–47]. Given these constraints, and the logistic limitations of budget, time and the number of people in each unit, we used the number of people available in a unit as more practical base criteria for stopping recruitment. With our purposive sampling, where the goal was to acquire data representative of information that would be typical of a setting instead of statistical significance, our recruitment and sampling strategies are intended to provide rich qualitative data. The data and findings, however should not be taken to represent the entire population given this is a purposive sample. Our goal was to acquire information from diverse roles on their perspectives, experiences and beliefs.

We aimed to achieve a representation of some types of workers, such as discharge navigators, who were present in low numbers in the patient care units. Some roles in our strata, such as the discharge navigators within each unit were oversampled because this sub-group is a smaller part of the population. With representation, our intent was to represent the “diverse” roles within each unit that provide patient care and administration. Our goal was to represent different strata of healthcare workers, who can provide meaningful and rich data.

Thirty-four healthcare workers (19 females, 15 males) participated in the study. Participants included five registered nurses, one nurse practitioner, one discharge navigator, 13 attending physicians, two residents, four fellows, two respiratory therapists, four social workers, and two clerks. Although clerks are typically not considered healthcare workers, they are a vital link in managing critical health information, and provide support services that influence the delivery of care; therefore, we included clerks in our study.

Participation in the study was voluntary, and participants could withdraw at any time. To avoid revealing their identities, we did not collect any specific demographic characteristics from participants when discussing their strategies, except gender. Our study focus was on the care process and not on individual characteristics of healthcare workers.

### Informed consent

The Institutional Review Board and the Nursing Research Review Committee approved the study. For in-person interviews, the investigator provided a copy of the consent document for the participant’s reference. In the case of telephone interviews, an electronic copy of the consent document was emailed to the participant. During the consent process, the investigator verbally reviewed the study with the participant in detail. Individuals interested in participating in the study provided verbal affirmation of consent. Written consent was waived to prevent linking of personal identifiers to the interview data.

### Interviews

The first author, who is trained in qualitative research, conducted 30- to 45-min telephone or face-to-face interviews with participants. We used both phone and in-person interviews to be flexible to healthcare workers’ schedules. Some healthcare workers and clerks were not available to participate in the interview during regular work hours. To ensure they were still able to share their perspectives, we utilized telephone interviews. A total of 9 participants were interviewed via phone. The rest of the 25 interviews were conducted in-person. The interviews included two open-ended questions: (1) What are your thoughts on readmissions? and (2) How does readmission change your process? The two specific questions on readmissions (see Additional file 1) reported in this paper were part of a larger research study on developing cognitive information models for healthcare workers. The interviewer also requested specific examples and sometimes restated responses to seek clarification about readmission processes and steps when needed.

Interviews averaged 34 min in length. Each interview was audio-recorded and transcribed using Transana™ (Transana™, Wisconsin) [48]. Transcripts from the audio excluded any identifying information.

### Coding template development

We used an inductive approach to data coding [41] and let the progression in analyses guide the development of themes about readmissions. In an inductive approach, the analyst approaches the data without any preconceived hypotheses or theoretical frameworks for the data [41]. Instead, analysis of specific observations is used to guide development of patterns and theory.

Categories, sub-categories and final themes were generated using the constant comparative framework of the grounded theory approach [49]. We therefore did not begin coding template development with preconceived themes; rather, the process of coding the data into different categories and subcategories, and continual review of these categories and subcategories led to themes emerging from the data [41] using the constant comparative method [49].

The first author trained the second author on each step required for coding. Training included discussions about qualitative analysis, and coding demonstration with a transcript. We began coding template development with an initial goal of independently reviewing approximately 25% of all data, and reached convergence when we evaluated 35% of all data. The two authors independently reviewed the same 12 randomly selected transcripts from the 34 study transcripts, about 35% of all data, to evaluate whether we were reaching convergence in codes; if we were not, our plan was to review more transcripts for the initial open coding. We then independently open coded the 12 transcripts iteratively to develop a coding template. Specifically, we reviewed participant responses to the two interview questions and coded the responses at the sentence level to generate text segments and the corresponding codes [50]. When reviewing the interview text sequentially we generated codes as they occurred in the text, and sometimes a sentence generated more than one unique code; at other times, an entire paragraph yielded only one code.

We repeated a code for a different text segment only if the context represented by the text was unique. After two consecutive iterative rounds of open coding all 12 transcripts, we reached consensus [50], and finalized a coding template for application to all 34 transcripts. To reach consensus, the original text in any text segment that was coded differently by the two authors was carefully reviewed and discussed in the context of the interview questions. We either agreed on a code that best represented the text, or generated a new code if the initial codes did not fit the text [50].

The coding template yielded four broad categories:

1. Causes of Readmission
2. Perception of Differences between Readmissions and Admissions
3. Steps Taken During Readmissions
4. Strategies for Preventing Future Readmissions

The broad category *Causes of Readmission* included four subcategories: *Patient Condition/Clinical, Care Consequences, Patient Education/Knowledge, Socioeconomic Status*.

See Table 1 for the complete categories, sub-categories, the 34 individual codes, and their definitions. Each sub-category for *Causes of Readmission* included nested individual codes. For example, the subcategory *Patient Clinical Condition* further includes the nested individual code *Patient Non-compliance with Treatment*.

**Table 1:** Template Indicating Study Codes and Definitions

Code Category	Definition
Causes of readmissions	
Patient/Clinical Condition	
Deteriorating Health (unpreventable)	Readmission due to worsening health; not necessarily preventable
Non-compliance with treatment	Readmission due to failure to follow instructions regarding medications, diet, etc.
Individual Characteristics	Readmission due to preference for care at hospital
Care	
Complications with Medication	Patient readmitted due to adverse reactions with prescribed medicine (from last admission).
Issues with Diagnosis	Patient readmitted due to missed or misdiagnosis or care during previous visit
Discharge Premature	Patient was discharged from care too early (sometimes due to prioritization needs in ICU)
Issues with Handoff to Primary Care Provider	Failure to contact/alert primary care provider about patient condition
Issues with Follow-up by Primary Care Provider	Follow up by primary care provider(s) not adequate
Education/Knowledge	
Patient not Educated Sufficiently	Patient readmitted due to inadequate education regarding self care after discharge
Socio-Economic Status	
Patient Lacking Access To Medication	Patient unable to obtain medications that would have prevented readmission
Patient Lacking Outside Social Support	Patient unable to obtain outside support, e.g., from friends or family
Perception of differences between readmissions and admissions	
More Knowledge/ Access to More Information	For readmissions the provider has access and an understanding of the previous medical history (i.e. why patient was previously admitted).
Shift in Information Management	Staff may not be as concerned about seeking care information because they know why the patient was readmitted (opposite of Degree of Assessment below).
Shift in Degree of Assessment	More conservative approach in assessing a readmitted patient, i.e., tendency to be more cautious in treatment.
Shift in Goals	Shift in focus to getting the patient stable enough to go home
Shift in Communication Needs	Perception of reduced need for comprehensive communication because initial communication among healthcare workers was established during original admission.
Shift in Need to Ask Protocol Admission Questions	Perception of reduced need to ask protocol admission questions; the responses are already on record.
Shift in Lab Work	Perception of reduced need for lab work; results from previous admission are available.
Waiving of Educational Requirements	The need to educate the patient is reduced; some education has already been provided.
Stigma Associated with Readmissions	A sense of disappointment by healthcare workers that they did not succeed in healing the patient;
Treating readmissions as new admissions	Considering every readmission as a new admission
Strategies for preventing future readmissions	
Educate the Patient	Future readmissions can be prevented by sufficient patient pre-discharge education regarding post discharge self-care
Follow Up with Patient After Discharge	Future readmissions can be prevented by calling the patients or scheduling patient visits after they are discharged
Improve Overall Care	Future readmissions can be prevented by improving care plan in hospital and overall care at home.
Steps taken during readmission process	
Identify Reason for Readmission	Look at/focus on why patient Is being readmitted, e.g. whether the same or new condition led to readmission
PSN (Patient Safety Net) Form	Fill out a PSN form indicating the reason for readmission
Insurance/Billing Steps or Considerations	Examine billing and insurance steps involved in readmissions to identify any concerns
Logging Documentation for Readmissions	Use the Electronic Medical Record (EMR) software for tasks such as patient data entry into the admission system for recording the readmission in the documentation system
Review Previous Care Records	Review information from the patient’s previous visit to the hospital
Team Communication	Communication among healthcare workers (e.g., nurses and doctors) about the readmitted patient
Obtain Feedback from Patient	Communicate with patient to obtain his/her perspective on the reason for readmission
Communicate with Primary Care Physician	Communicate with the primary care physician of the patient for his/her perspective on the reason for readmission
Improve Support System	Provide better support for post discharge care
Reconcile Medications	Recognize that the type or dosage of medication may need adjustment

Subcategories did not emerge from the other three broad categories; only individual codes resulted from the coding process for *Perception Differences, Steps Taken during Readmission,* and *Strategies for Readmission Prevention*.

When applying the code template to the 178 interview text segments contained in the 34 transcripts using Atlas.ti™ (Atlas.ti Scientific Software Development GmbH, Germany) [51], we examined each sentence in the transcript and followed the same process used for open coding.

### Conceptual networks among codes

We used Atlas.ti™ to develop conceptual networks from the generated codes containing themes and their relationships. When codes are examined for contextual relationships and linked, they produce networks. Networks can help visualize the data structure and attach meaning to codes [52].

In a network, a connecting line between the codes, and a label on the line, visually represent a relationship link. Links can be one-way, two-way or non-directional [52]. Labels on the relationship link represent how codes relate based on broad themes identified by the analyst from the raw data; for example, we labeled the relationship between the two codes “identify reason for readmission” and “shift in degree of assessment” as “results in” with a one-way directional arrow from “identify reason for readmission” to “shift in degree of assessment”. This was because the text segments from the raw data suggested that healthcare workers became cautious when the reason for a patient’s readmissions signaled an action they needed to take.

In our study, we developed the network diagram in two steps. In the first step, we created a diagram with all 34 codes (represented in boxes) but without any of the network linkages. Then, we focused on each code in the diagram and systematically considered the relationship of that code with all the other 33 codes in the diagram. We then linked the codes, and labeled the relationships. This process was repeated for all the 34 codes in the diagram. The constant comparative approach in grounded theory [49] was also employed in development of the conceptual networks as the links, relationships were iteratively examined. This approach in developing the conceptual networks helped form interesting themes about the underlying data.

In the second step, we focused on each code and its associated subnetwork, and re-evaluated whether the relationships and linkages identified in the first step were accurately represented. Refinements, including addition of any missed relationships from the larger network diagram, deletion of any relationships that did not make sense, and relabeling of relationship labels, were also made in the smaller networks.

The larger network diagram helped the analyst obtain an at-a-glance (forest) view of the entire data. The smaller network diagrams helped the analyst focus (tree view) and fine-tune the relationships among the major themes in the data.

## Results

Transcript analysis yielded qualitative insights on healthcare worker strategies and processes on readmissions, and on differences from regular admissions.

Conceptual Networks for Provider Strategies on Readmissions.

We aimed to document shifts that healthcare workers make to their strategies and processes when treating readmitted patients. Three major themes were identified from eleven smaller conceptual networks. The themes and the associated smaller conceptual networks are presented in the following sections, with representative verbatim quotes from participants, wherever necessary, to relate the data to the themes.

### Theme 1: Shifts in clinical assessment

**Fig. 1:**
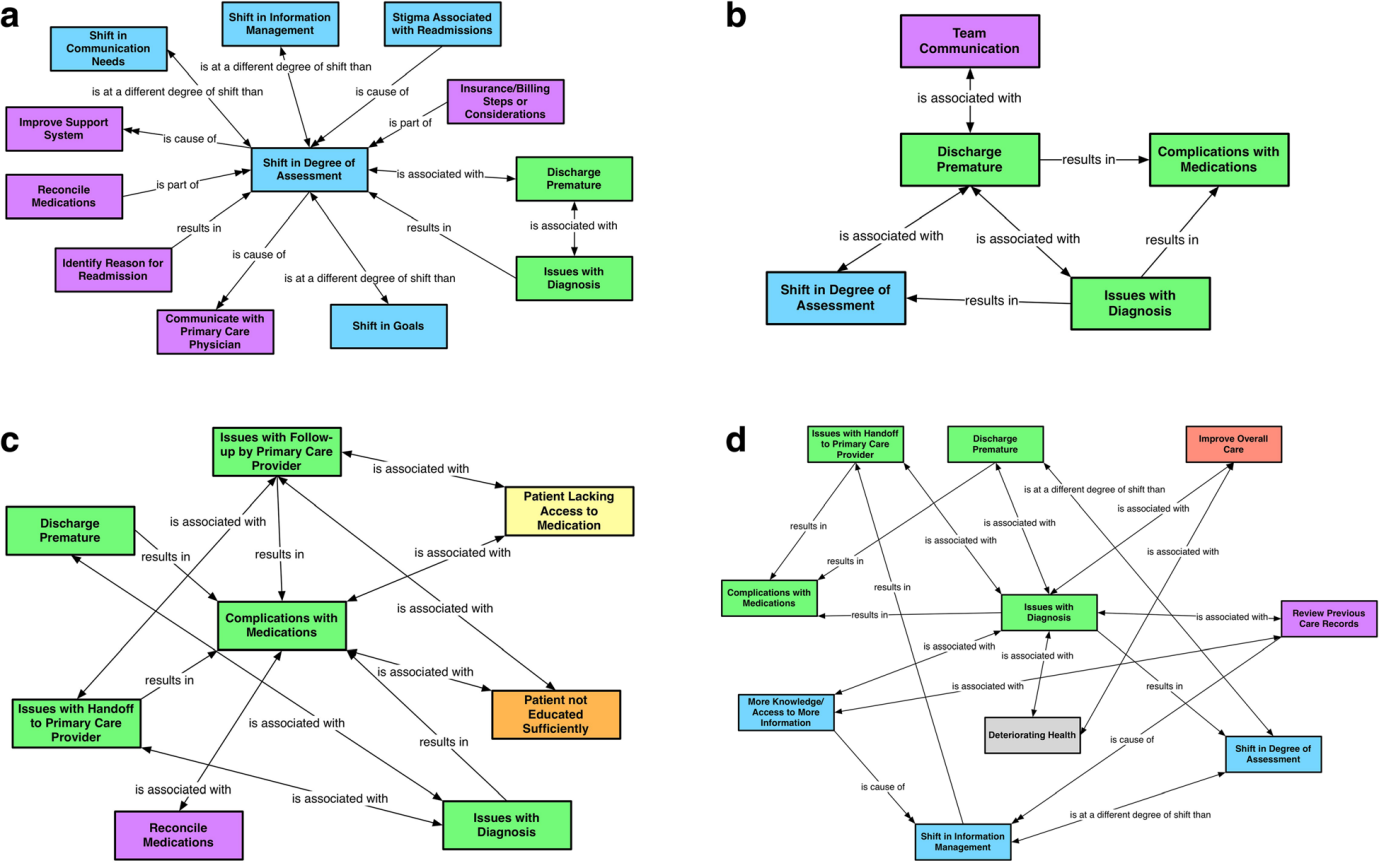
**a, b, c, d** Theme 1: Shifts in Assessment. Each of the figures represents a conceptual network with one code as the focal code (always in the center of the figure), and relationships to other codes shown. These four figures or conceptual networks together represent the unifying theme of shifts in assessment. The four focal codes used in the four networks are shift in degree of assessment, discharge premature, issues with diagnosis, and complications with medications.Applies to all figures from this point forward: Text bubbles represent the code categories. Color codes for causes of readmission: green, care; orange, education/knowledge; grey, patient/clinical condition; yellow, socioeconomic status. Blue indicates perception of differences between readmissions and admissions. Purple indicates steps taken during the readmission process. Red indicates strategies for preventing future readmissions. One-way relationships are indicated by a unidirectional arrow, and two-way relationships are indicated by a bidirectional arrow. Causal relationships are illustrated by a double-headed arrow. The labels on the lines represent the relationships between the codes. The labels used are: Is a different degree of shift than; Is associated with; Is a; Is cause of; Is part of; Is property of; and Results in

When assessing a readmitted patient’s clinical condition, healthcare workers increased the assessment quantity and depth. As illustrated by the following sample quote from a participant in our study, healthcare workers became more conservative in their assessments (Fig. 1a).

*“I think that always makes you reassess the patient a little closer and be more cautious about sending him out again.”*

When a patient was readmitted, healthcare workers felt intense scrutiny (Fig. 1a). They then became more cautious in caring for the readmitted patient, particularly if the patient had been discharged early or if they thought their initial care was inadequate (Fig. 1b). If the care team discharges a patient early, they can miss the patient’s critical clinical indicators that might otherwise delay discharge. A decision to discharge early can eventually cause complications with either medications or clinical condition, resulting in a readmission, as the following quote and Fig. 1c suggest.

*“Sometimes I think the patients are let go before they are ready.”*

*Maybe sometimes the patients are discharged earlier than they should have been, for example if the patient wants to go or things are not completed, they get readmitted again.*

*“Or was there something that we missed, frankly…we didn’t get the right diagnosis the first time.”*

However, when readmitting a patient because discharge was premature (Fig. 1a and b), healthcare workers tended to become more conservative in assessing the appropriate discharge time. Additionally, if they missed an important diagnosis during the first admission, healthcare workers became even more guarded in their assessment (see the following quote and Fig. 1d).

*“Sending the patient out, it may have an impact because of the fact that…the criteria may change a little bit…We now realize that the patient came back once already. So we try to intervene and make sure that this patient is optimized much more than we would do for any other patient, just because…that patient would end up coming [back]. We don’t want the patient to come back again… I think we are more cautious about sending them out again.”*

### Theme 2: Shifts in use and management of information

**Fig. 2:**
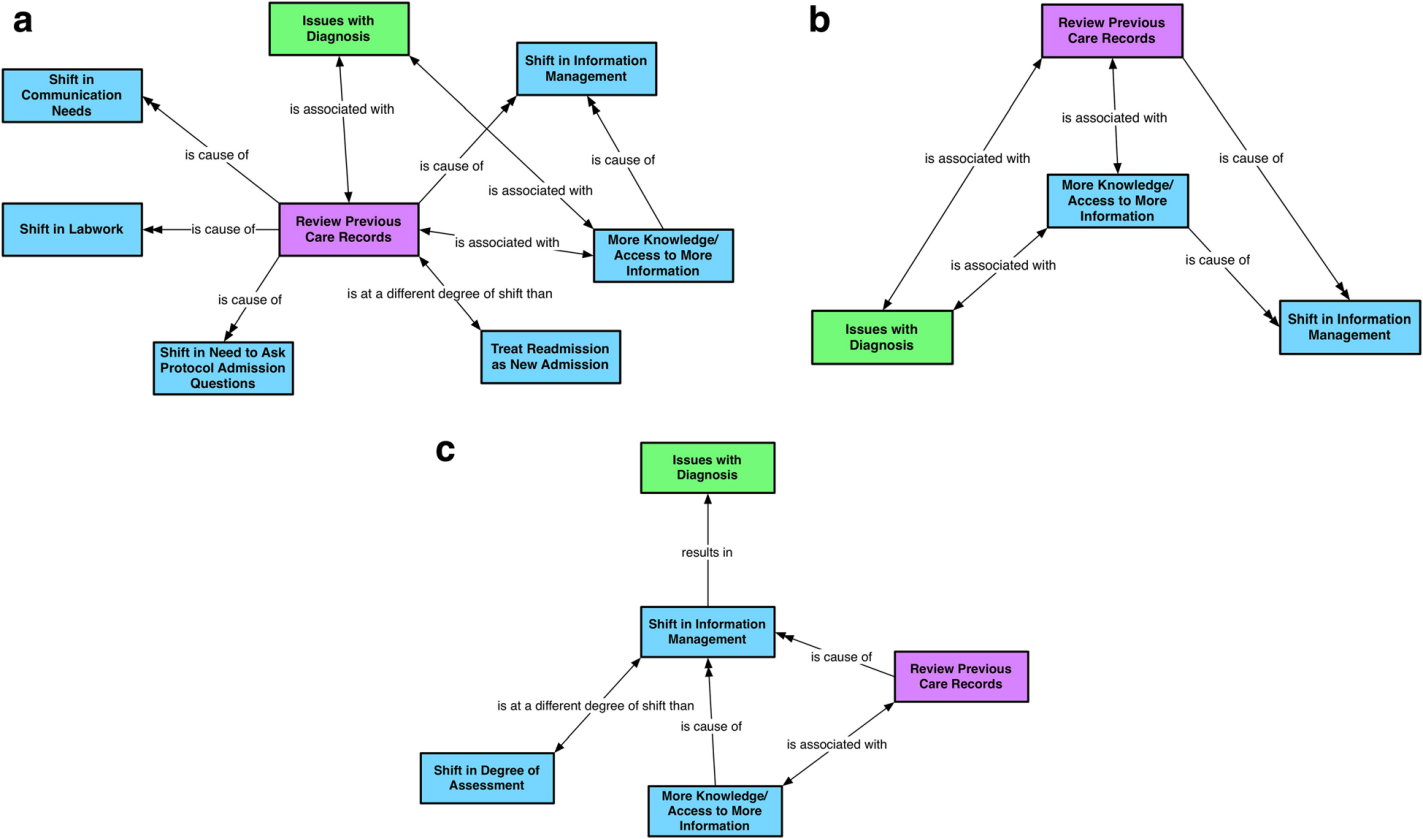
**a, b, c** Theme 2: Shifts in information management. Represents the theme emerging from three conceptual networks, with focal codes shift in information management, more knowledge and access to information, and review previous care records

Our findings indicate that during readmissions, healthcare workers review available information about the patient from their past and most recent visit as part of the conservative shift in assessment discussed earlier (Fig. 2a). Compared with a regular admission, access to past information about the patient was reported as a major difference in a readmission. (Fig. 2b). Reviewing past records also enables workers to plan lab work for the patient, eliminating any tests that have already been done (Fig. 2a).

Healthcare workers may rely on prior information for use in their clinical decisions. (see the following quotes and Fig. 2c). Heavy reliance on prior information has the potential to influence how diagnosis decisions are made (Fig. 2b).

*“Because the patient is familiar to us, definitely…we will have a different viewpoint of a patient who is newly admitted because we know the patient well and we will continue the care that we gave.”*

*“You have past medical history and all that [already] in the records, and what was just recently done to the patient.”*

Additionally, because prior information about a patient is available, healthcare workers may not feel the need to be as detailed in their information gathering and documentation during readmission, indicating a shift in their information management, as the following quotes and Fig. 2c indicate.

*“The process might be a little easier in terms of you know, not having to go back in the charts in a very detailed way.”*

*“And so I think it makes the communication…flow better when we have documented correctly what we have already done. And that way we don't have to…redo everything. We just kind of start at where it was left off when the patient left here.”*

*“So when you have a readmission, I think the only thing that changes, that makes it different, is if the patient is coming back with very similar to what they were discharged with, so then you have like part of their past medical history, and all that on the electronic records.”*

### Theme 3: Shifts in communication patterns

**Fig. 3:**
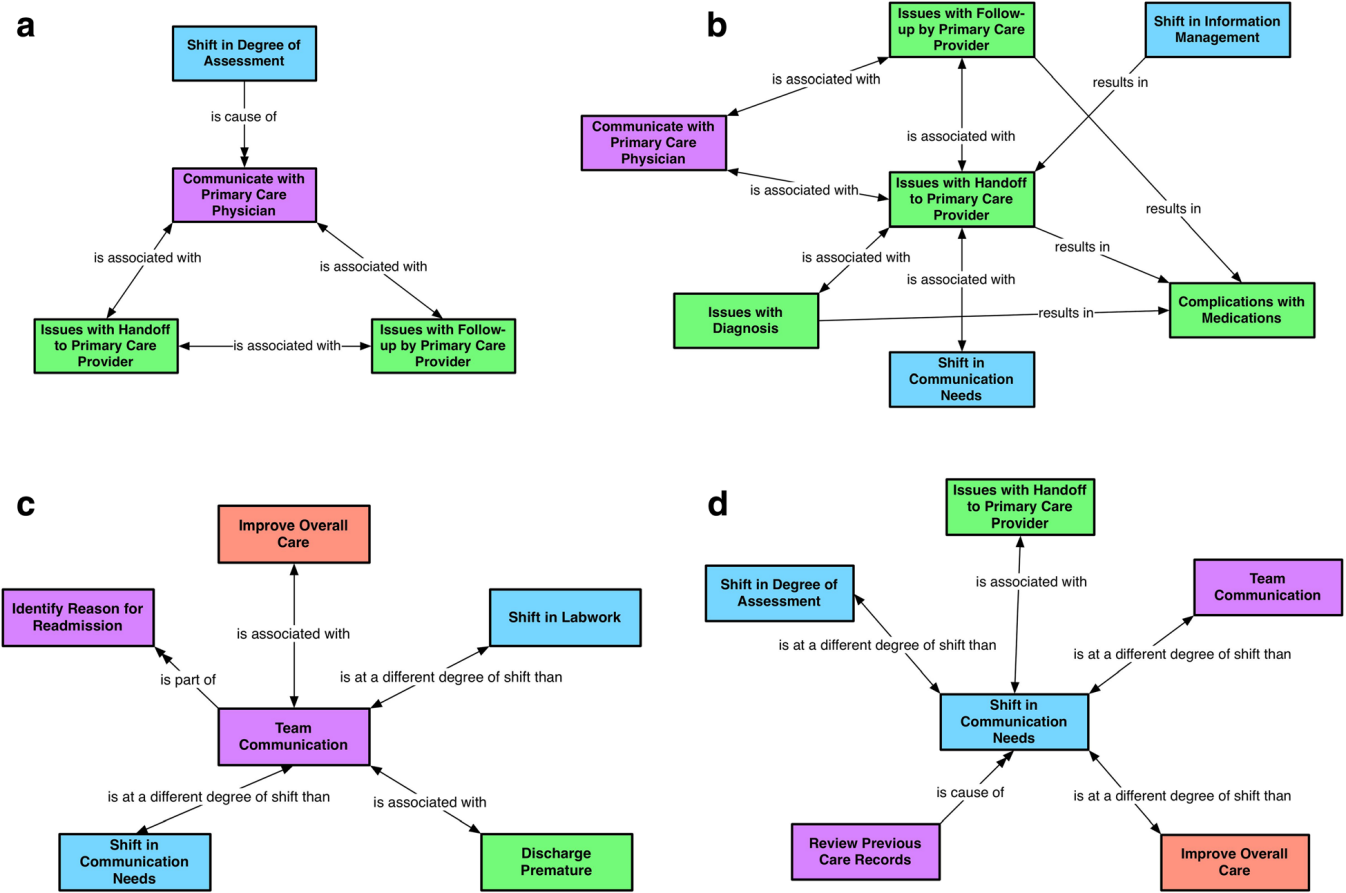
**a, b, c, d** Theme 3: Shifts in communication patterns. Represents the theme emerging from four conceptual networks with focal codes communicate with primary care physician, shift in communication needs, team communication and issues with hand off to the primary care provider

As part of the increased caution in their clinical assessments, healthcare workers suggested enhanced communication with the patient’s primary care physician (Fig. 3a), as indicated by the following quote from a participant who reported that they would address concerns for a readmitted patient by targeting communication with the primary care physician.

*“did we fail to handoff to their primary care provider and when they came back, they saw some laboratory abnormality and thought that the patient need to be readmitted, when they are [admitted]….And we try to assess that and then detail our education towards that problem right. And so, be it more patient education, be it better handoff from physician to physician……we try to target those things.”*

Improved communication with primary care providers can prevent inadequate handoff; effective handoff can enable the primary care provider to provide adequate follow-up care and education to the patient after discharge (Fig. 3b).

Some healthcare workers also reported a need for increased communication among team members (Fig. 3c), while others did not feel the need to communicate as much because they had always had access to the patient’s chart and could look at all the previous patient history and care information (Fig. 3d), as indicated by this quote from a participant:

*“…and then report may not be as extensive just because the unit may know the patients already…so it’s a little bit easier, they can just tell you the changes that have occurred in the last 12, 24, 48 hours.”*

## Discussion

In our study, we aimed to document shifts that healthcare workers make to their care strategies when dealing with readmitted patients. Three main findings emerge from our study:

1. Healthcare workers shift their thinking and actions when clinically assessing readmitted patients: they become more conservative and cautious in their clinical assessments;
2. Healthcare workers extend their conservative approach to care, by consulting, and in many cases relying on existing information, about a readmitted patient, but may not necessarily have the need to be detailed in their new documentation to guide their clinical assessments;
3. Healthcare workers shift their communication patterns. They report a need for more internal team communication and communication with the primary care physician. At the same time, they report that they do not feel the need to extensively communicate with other departments within the hospital because of the availability of past information about the patient to all departments that share the common information system. We discuss each of these three findings in turn.

### Shift towards conservative clinical assessments

Our study findings indicate that healthcare workers become conservative in their clinical decisions, and shift their care strategies when treating a readmitted patient. We think healthcare workers become cautious with a readmitted patient for two main reasons: (1) they might consider a readmission as a failure of care during the first admission that they wish to correct; and (2) in many cases, patient clinical conditions also trigger readmissions, and healthcare workers flag such cases as high-risk for repeated admissions. They may therefore perceive an elevated risk for a readmitted patient and adopt a more cautious treatment approach.

Research in decision-making [53, 54] supports our interpretation that people generally become cautious when they perceive a high-risk potential for failure, and when they want to correct past mistakes. According to choice shift theory [53, 54], people tend to become more risk-averse when payoffs for others are at stake; they become less risk-averse when the payoffs only affect them directly [55]. In the case of the healthcare workers, given that they are acutely aware of the risk and consequences for their patients if they make mistakes, they are likely to be risk-averse and cautious.

A second interpretation of healthcare workers’ conservative behavior is based on the literature on compensatory behavior [56] as a protective mechanism when one is confronted with risks from failures and errors. Research, predominantly conducted in the transportation safety domain, indicates that people become less risk-averse (and are inclined to risk more) when they feel safe and protected (e.g., with use of safety seat belts); conversely, they become more risk-averse (and take less risk) when they perceive a higher level of risk and lack protection [56, 57]. Our thinking is that healthcare workers may be seeking protection from further risk during readmission episodes by compensating with a cautious approach.

A third plausible explanation for the conservative behavior exhibited by healthcare workers is self-correction. In high-risk, high-consequence systems, such as aviation crew resource management, self-correction from feedback has been demonstrated to be an essential process for maintaining system integrity [58]. Self-correction mainly occurs by learning about the past, adjusting one’s current goals and actions based on knowledge about the past, and monitoring the present to prevent any errors. When healthcare workers self-correct, they might trigger a shift in their decisions and actions based on the shared feedback and learning.

It is debatable whether adopting a conservative approach is better than approaching the readmitted patient as a new admission. The influence of biases in judgment and decision making, although well understood in medical diagnosis [59], has not been explored in the context of readmissions. Several important questions arise regarding biases in relation to behavioral shifts in healthcare workers. If healthcare workers become cautious, are they heavily influenced by what happened in the past? Would this affect their ability to form new perspectives about the patient’s condition?

### Shifts in information use

Our findings suggest that because information about the patient is already available in the system, healthcare workers tend to rely on that information. A majority of healthcare workers in our study reported reviewing past information about the patient to ensure a correct course of action, and to refresh their memory about the patient. However, healthcare workers also reported that they did not need to make detailed notes when a patient was readmitted compared to when a patient was newly admitted. We speculate that because healthcare workers’ rely on readmitted patients’ past information, and express their lack of need for creating new detailed notes every time there is a readmission, they may not be immediately aware that care decisions and actions are highly dependent on the information documented in the systems [60].

In many hospital systems, documentation is often considered an administrative burden that is time consuming and challenging to maintain on a daily basis [61, 62]. Hence, healthcare workers, often unintentionally, consider documentation as an activity separate from treatment that needs to be completed for administrative purposes [63]. Given the challenges of creating new documentation, it is conceivable that one would use documentation that is readily available about the patient.

When recent past information about the patient exists, then it is conceivable that healthcare workers may be naturally inclined to use the available information. This behavior may be an instance of the well-documented availability bias. We know from the theory on heuristics and biases [64], and particularly from the availability bias literature [59, 65], that using past and more recent information can bias an individual to make decisions based only or heavily on that information. However, if healthcare workers were to treat readmitted patients like new admissions, would they miss important clues from the past? Would they generate care decisions and actions that are novel compared with the previous admission?

Healthcare workers might rely on past information as a means of self-correction and error prevention mechanisms [66], with the belief that they would be better informed about the readmitted patient using clear documentation about past treatment decisions and actions.

Healthcare workers’ reliance on past documentation in the information system also suggests an over-reliance phenomenon. Over-reliance on any automation indicates a tendency to trust the automation to be correct more than is warranted. Research in aviation [67], and more recently in healthcare systems [68], has concluded that over-reliance on automation can lead to complacency and errors [69].

Design of the information system, and the extent of technology support, may also affect the extent of evaluation healthcare workers may exercise when managing information about patients. The way documentation policies and systems are designed [70], for instance, can discourage healthcare workers from engaging in a thorough review of the information about a readmitted patient. Additionally and paradoxically, administrative pressures to perform well and to improve patient care [61, 62] may undercut any efforts to improve information related to the patient.

### Shifts in communication patterns

Our findings suggest shifts in healthcare workers’ communication patterns that closely align both with their shifts in clinical assessment procedures for readmitted patients and with the changes in information management discussed above. Healthcare workers report a need for increased team communication when a patient is readmitted. Additionally, they report that they enhance their communication with the primary care physician to prevent future readmissions. The caution they exercise in communication practices aligns with their shift toward becoming conservative and cautious in clinical assessment. Increasing the frequency of communication among team members is one strategy they use. The reported increase in communication may also be a reflection of their self-correction behavior [66], as discussed earlier. Healthcare workers may also become increasingly aware of communication problems in the system during their routine training, because of the widespread recognition of communication-related patient safety consequences. Therefore, they may be readjusting their communication practices to improve their clinical assessments.

Our findings, however, indicate discrepancies in their communication patterns when dealing with information access and use. Some healthcare workers report that they do not need to be as extensive in their communication, and that their communication flow will be easier for a readmitted patient given the availability of past information about the patient. Similar to the shifts we observed in information access and use, healthcare workers may not feel the need to communicate extensively because of the ready availability of prior documentation about the patient.

In summary, while the healthcare workers strive to enhance their communication practices as part of their conservative shifts, they also report a decrease in the extent to which they need to communicate, indicating individual variations in these communication patterns. Healthcare workers may be increasing their communication practices in one aspect of their care, and concurrently reducing the details and quantity in other aspects of care, depending on how they prioritize care tasks for a readmitted patient. These variations in communication patterns need further research, given the importance of communication in enhancing patient safety and preventing readmissions.

### Study limitations

We purposively sampled typical provider roles in the hospital. While richly representing multiple perspectives on readmissions, we did not seek input from some roles, such as hospital administration. While the second interview question may have potentially prompted the participants to think about changes in the readmission process without first examining if there is one, the question is not leading, because the entire tone of the interview was open-ended with follow-up questions, and participants were free to say that nothing changed in their process when a patient was readmitted. A few participants prefaced their response by saying “while nothing else about the process changed….”. The open-ended nature of the entire interview facilitated participants in providing in-depth feedback. Additionally, our question was aimed towards obtaining feedback on the “how”, which if not present, the participants would mention as no shift in the process.

An inquiry perspective guided the generation of relationships in the conceptual networks, so we could generate new theory about provider actions and behavior when readmitting patients. We used the raw data to generate visual representations for examining critical relationships. This method does not allow inferences about temporality or causality. For example, inquiring whether patient non-compliance relates to insufficient education about the medications does not conclusively establish a relationship between these variables.

We structured the steps in interviews and in analyses to reduce investigator bias. The authors, as outsiders, were not privy to unit-specific information. When coding, we did not measure inter-rater reliability because we finalized the coding template based on consensus [50]. However, the iterative and consensus-based open coding process helped reduce bias.

### Study implications and future work

The findings from our study suggest further investigation into shifts in thinking and behavior among healthcare workers. These shifts are important to understand because they directly influence patient care decisions and actions, and are often not readily apparent. Understanding these shifts in thinking and behavior also provides opportunities for the team and/or organization to intervene in a timely manner, either to make course corrections or to learn best practices for the future. Lessons and best practices from making these shifts can be shared and sustained in knowledge management and organizational learning systems to promote error prevention.

Currently, provider learning is often siloed within their teams, and only informally discussed outside the teams. While many hospitals have structured mechanisms (i.e., meetings, huddles) to discuss patient safety problems, these mechanisms may not focus on what can be learned and shared from readmissions.

The most important implication from our study is the potential consequences of the shift in information management to patient safety, knowledge transfers and the need for better design support to enhance documentation systems. A problem with using existing information is that it is almost impossible for healthcare workers to know if there are inconsistencies or problems in that information. Due to this invisibility of inconsistencies and errors in the information, healthcare workers may inadvertently continue to use and communicate potentially outdated or erroneous information. Given the volume of patient information documented during every visit, identifying prior erroneous information contributing to readmissions can challenge even the most seasoned healthcare workers.

Additionally, documented patient information may not always facilitate the thinking required for effective treatment. Although healthcare workers document “what” they did for a patient throughout the patient’s stay, they may not comprehensively document “why” they did what they did. The why is part of their implicit knowledge obtained from their assessment and interaction with the patient. Increased assessment for a readmitted patient does not necessarily translate into improved documentation.

Even when good documentation is readily available, it may not be usable [71]. The interface may make it difficult for healthcare workers to differentiate and highlight information specific to a readmission episode. Using the same interface for regular admissions and readmissions not only adds to the volume of information for a patient, but masks important new information under what may already be known about a patient.

## Conclusions

The findings from our study indicates shifts in provider strategies when managing readmitted patients. Healthcare workers may exhibit a tendency to become more conservative with readmissions. However, readily available patient information from the previous admission played a large part in guiding their thinking. A more conservative approach with a readmitted patient, on its own, does not necessarily lead to improved documentation or better patient care. Understanding the relationships between provider experiences and reasoning, patient-care activities, and information systems design can make healthcare delivery effective.

## Additional file

Additional file 1: Interview Guide. Contains semi-structured interview questions used in this study. Only questions that directly relate to the manuscript’s research goals are provided. Other interview questions that were a part of a larger study are not included. (DOCX 51 kb)

## Abbreviations

ICU: Intensive Care Unit
US: United States
