# Supplementary material for: A qualitative investigation of healthcare workers’ strategies in response to readmissions
Source: BMC Health Serv Res. 2018 Feb 27;18:138. doi: 10.1186/s12913-018-2945-9 (PMC5827983; doi:10.1186/s12913-018-2945-9)
Supplement: Supplementary file 1 — Interview Guide. Contains semi-structured interview questions used in this study. Only questions that directly relate to the manuscript’s research goals are provided. Other interview questions that were a part of a larger study are not included. (DOCX 51 kb) [file 12913_2018_2945_MOESM1_ESM.docx]

**INTERVIEW GUIDE**

Topics of discussion include:

(a) inputs and outputs of the discharge process

(b) major steps, stages or events that each typical patient goes through in every unit to progress in their care and ultimately get discharged, and have follow up.

(c) boundaries of the system including any time limitations, stakeholders in the process, organizational and structural boundaries.

(d) tools, technologies and information that is typically used to support the major process steps

(e) suggestions for improvement of the process

1. What are your thoughts on readmissions?
2. How does it change the process steps you just described?
